# A novel chimeric lysin with robust antibacterial activity against planktonic and biofilm methicillin-resistant *Staphylococcus aureus*

**DOI:** 10.1038/srep40182

**Published:** 2017-01-09

**Authors:** Hang Yang, Huaidong Zhang, Jing Wang, Junping Yu, Hongping Wei

**Affiliations:** 1 Key Laboratory of Special Pathogens and Biosafety, Center for Emerging Infectious Diseases, Wuhan Institute of Virology, Chinese Academy of Sciences, Wuhan 430071, China

## Abstract

Methicillin-resistant *Staphylococcus aureus* (MRSA) is one of the most threatening pathogens due to its multi-drug resistance (MDR) and strong biofilm-forming capacity. Here, we described the screening of a novel chimeolysin (ClyF) that was active against planktonic and biofilm MRSA. Biochemical tests showed that ClyF was active against all *S. aureus* clinical isolates tested under planktonic and biofilm conditions. Structure analysis revealed that ClyF has an enhanced thermostability and pH tolerance than its parental lysin Pc by forming a hydrophobic cleft in the catalytic domain and an Ig-like structure in the cell-wall binding domain. A single intraperitoneally or topically administration of ClyF showed good MRSA removing efficacy in mouse models of bacteremia and burn wound infection, respectively. Our data collectively demonstrated that ClyF has good bactericidal activity against planktonic and biofilm MRSA both *in vitro* and *in vivo*, and therefore represents a useful antibacterial to combat MDR *S. aureus*.

*Staphylococcus aureus* is an opportunistic pathogen that commonly colonizes nasopharynx and skin^1^, however, most of these strains have the capacity to cause severe infections when getting access to wounds and soft tissues, even progress to life-threatening diseases such as pneumonia or necrotizing fasciitis^2^. Due to its strong biofilm formation capacity and extensive antimicrobial resistance^3^, methicillin-resistant *S. aureus* (MRSA) biofilm-associated infections are more difficult to treat with antibiotics^4^. This is significant because *S. aureus* cells within biofilms can be a hundred to thousands of times more tolerant to common antibiotics^5^. MRSA biofilms are thus an important contributing factor for many treatment failures^6^, persistent infections^7^, increased risk of replacement of medical devices^8^, and the morbidity and mortality of most MRSA associated infectious diseases^9^.

Lysins are phage encoded cell-wall hydrolases that cleave the bacterial peptidoglycan for the release of progeny virions^10^. Because of their potent species-specific activities and rare bacterial resistance reported, lysins have received particular attention as promising alternatives to antibiotics^11^,^12^,^13^. In the last decade, several staphylococci targeted lysins have been developed and examined in experimental animal models to validate their efficacy as therapeutic agents^14^. However, natural staphylococcal lysins frequently suffer from solubility/stability and activity problems^15^,^16^. Moreover, the activities of some staphylococcal lysins are evidenced to be affected by working conditions, such as milk^17^, and ions concentrations^18^.

Natural lysins from phages infecting Gram-positive bacteria have a modular structure, that is, an N-terminal catalytic domain (CD) and a C-terminal cell-wall binding domain (CBD). Developing chimeric lysins (chimeolysins) by shuffling these two domains from different natural lysins has been demonstrated to be a promising strategy for developing novel antibacterials with improved properties^12^, such as enhanced activity^19^, improved solubility^16^, extended host range^20^, and intracellular killing capacity^21^. However, the current trial-and-error approach for identifying ideal chimeolysins is inefficient, making new strategies imperative. Our previous study has described an induced lysis-based screening method and its application in rapid screening of chimeolysins against streptococci^22^. This method is based on finding active bactericidal chimeolysins from a chimeolysin library with different combinations of CDs and CBDs expressed in *E. coli* by checking clear zones on culture plates. The chimeolysins inside *E. coli* cells were released by the aid of a novel enzyme, ClyN, which can cause controlled lysis of *E. coli* cells.

In the present study, a novel staphylolytic chimeolysin, ClyF, was identified using the screening method, and its bactericidal activity against planktonic and biofilm MRSA was further ascertained *in vitro* and *in vivo* in mouse models of bacteremia and burn wound infection.

## Results

### Screening of chimeolysins against staphylococci

To identify chimeolysins active against *S. aureus*, a library of 126 chimeolysin clones representing chimeras of a random combination of the seven CDs and the three CBDs were screened for the ability to form large, clear lysis zones in a lawn of *S. aureus* N315. One clone, AK114, was distinguished by the production of the largest clear zone in the lawn of the target bacterium (Supplementary Fig. S1). Sequencing analysis showed that the chimeolysin produced by the clone AK114 is consisted of the CD from the Ply187 lysin (Pc, the N-terminal 157 amino acids) and the CBD from the PlySs2 lysin (PlySb, the C-terminal 99 amino acids), and this chimeolysin is named as ClyF for short. Because ClyF showed the highest activity on plates, its characteristics were further studied.

### Characteristics of ClyF

**Figure 1:**
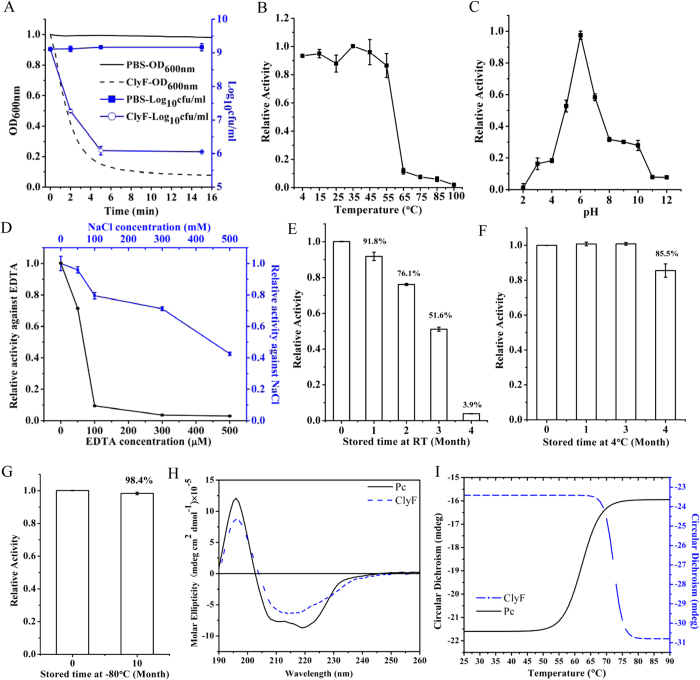
Characteristic of ClyF. (**A**) Time-killing curves of ClyF against *S. aureus. S. aureus* N315 were washed once with PBS and then treated with either 25 μg/ml ClyF, or equivalent volume of PBS buffer only, the changes of OD_600nm_ were monitored by a microplate reader at 37 °C for 15 min (left-Y axis, blank lines). Meanwhile, the viable cell number was calculated by plating onto TSB agar plates at various times (0, 2, 5, and 15 min, right-Y axis, blue lines). The effect of temperature (**B**), pH (**C**), EDTA (left Y-axis in (**D**), blank line) and NaCl (right Y-axis in (**D**), blue line) on the lytic activity of ClyF. The lytic activity of ClyF in each condition was compared and normalized as activity relative to the maximal activity. (**E**–**G**) Storage stability of ClyF. ClyF was stored at RT (**E**), 4 °C (**F**) and −80 °C (**G**) for different times (0–10 months). The residual activity against *S. aureus* N315 was determined by a microplate reader at 37 °C for 15 min, related to the activity of the fresh ClyF before storage. (**H**) Circular dichroism spectra of ClyF and Pc. ClyF (7.7 μM) and Pc (10.7 μM) in 4 mM Tris-HCl (pH 7.4) were scanned by a circular dichroism spectrometer from 190–260 nm at RT. (**I**) Thermal denaturation curves of ClyF and Pc. ClyF (38.5 μM) and Pc (21.4 μM) in 4 mM Tris-HCl (pH 7.4) were monitored by a circular dichroism spectrometer at 220 nm at temperatures from 25–90 °C. The Tm of each protein is estimated by the instrument’s software. Groups treated with PBS were used as controls, each assay was repeated for at least three times. Mean values are plotted, and error bars represent 1× standard deviation.

Initially, we tested the chemical and enzymatic characteristics of ClyF. ClyF was expressed by *E. coli* as a soluble protein after induced overnight at 16 °C (Supplementary Fig. S2A), and after purification by Ni-NTA affinity chromatography a purity of >95% was obtained as shown by 12% SDS-PAGE gel (Supplementary Fig. S2B). As shown in Fig. 1A, ClyF was highly active against *S. aureus*, decreasing the turbidity of *S. aureus* N315 from 1.0 to near 0.1 within 5 min after treatment with 25 μg/ml ClyF, and leading to a reduction of 3.1 log10 in viable cell number.

Next, we tested the effects of temperature, pH, EDTA and NaCl on the lytic activity of ClyF in order to understand the working conditions of ClyF. Results showed that ClyF maintained over 85% activity after 1 h exposure to 55 °C and totally lost its activity after 1 h at 65 °C (Fig. 1B). The comparison tests of ClyF activity in buffers with different pHs showed that the pH tolerance range for ClyF was 4–10 (with a maximal activity at pH 6) (Fig. 1C), broader than that of Pc (which shows a pH range of 4–7, Supplementary Fig. S3). Further tests showed that EDTA (left-Y in Fig. 1D) and NaCl (right-Y in Fig. 1D) influenced the lytic activity of ClyF in a dose-dependent manner. Furthermore, we tested the storage stability of ClyF in various conditions. Our results showed that ClyF maintains 91.8%, 85.5% and 98.4% activity after being stored at room temperature (RT) for 1 month (Fig. 1E), 4 °C for 4 months (Fig. 1F), and −80 °C for 10 months (Fig. 1G), respectively. Notably, the lytic activity of ClyF was decreased drastically over the time period when stored at RT, nearly abolished after being stored for 4 months (Fig. 1E).

### Secondary structure of ClyF

In order to assess whether PlySb enhanced the conformational stability of ClyF, we compared the secondary structures and melting temperature (Tm) of ClyF and Pc. As shown in Fig. 1H, the circular dichroism spectrum of ClyF looks smoother in lineshape compared to its parental lysin Pc. The secondary structure evaluation results indicated that the α-helix content of ClyF decreases slightly (8.2% in ClyF vs 17.7% in Pc), and the antiparallel content increases largely (46.5% in ClyF vs 22.3% in Pc) (Supplementary Table S3), corresponding to the fusion of PlySb. The thermal stability of ClyF and Pc was then assessed by following changes in the circular dichroism spectra in response to increasing temperature (Fig. 1I). Results showed that the Tm of ClyF and Pc was 65.03 ± 0.28 °C and 55.73 ± 0.18 °C, respectively, suggesting that the PlySb binding domain enhances the thermal stability of ClyF.

### 3D structural analysis of ClyF

The BLAST results showed that Pc was the member of NLPC_P60 superfamily and the CBD of ClyF belongs to the member of SH3 superfamily. To understand the structure difference between Pc and ClyF, we performed molecular modelling in silico. The secondary structure comparison performed with Dali server indicated that Pc/ClyF structures were similar to lysin LysK from *S. aureus* bacteriophage K. The 3D models of Pc/ClyF were constructed by Rosetta3.5^23^ to further investigate how the CBD improved the Tm and pH tolerance of ClyF. These models fitted very well and provided us putative detailed interactions between the CD and the CBD.

**Figure 2:**
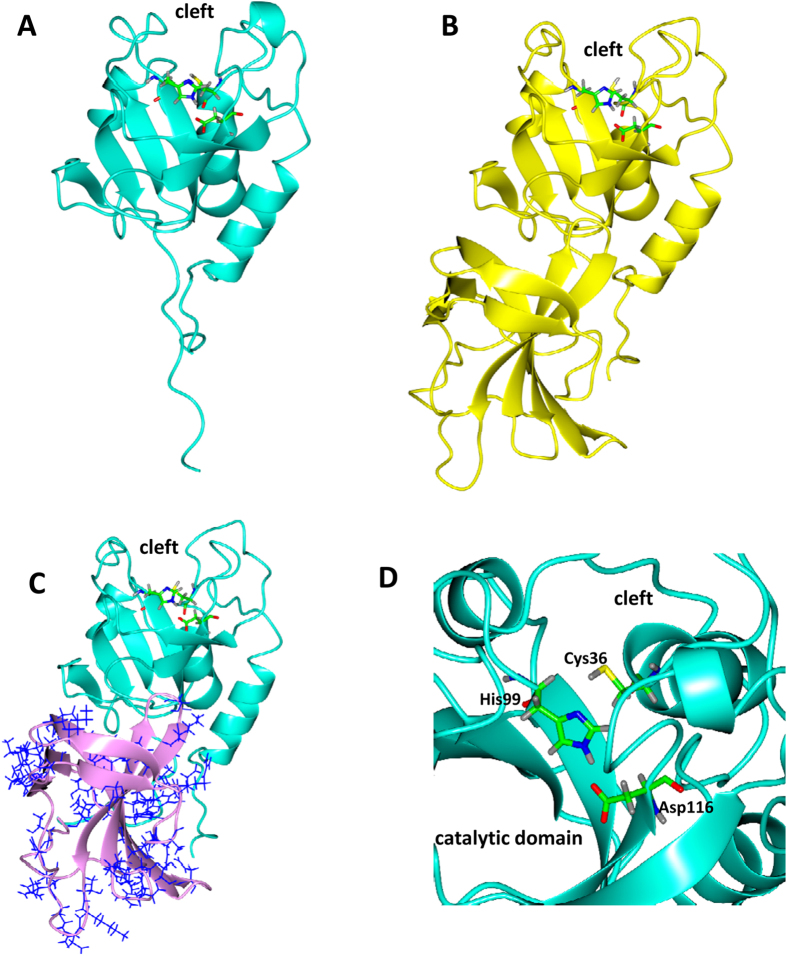
Structures of Pc and ClyF. (**A**) Overall structure of Pc (in ribbons) and the catalytic residues (in cylinders). (**B**) Overall structure of ClyF (in ribbons) and its catalytic residues (in cylinders). (**C**) Structure of the CD (cyan) and the CBD (pink) of ClyF with the hydrophilic residues of the CBD shown in bonds and colored in blue. (**D**) Close-up view of the conserved catalytic residues in the catalytic domain close to hydrophobic cleft in Pc.

The structure of Pc revealed a papain-like topology with a hydrophobic cleft, where the catalytic triad is located and act on peptidoglycan substrate (Fig. 2A). The structure of ClyF contains both the CD and the CBD showed in an Ig-like structure (Fig. 2B). In ClyF, the occupancy of α-helices and strands is higher than Pc (Fig. 2C). Moreover, on the surface of the CBD there are almost hydrophilic residues, such as Glu, Asp, Lys, Arg, and His (Fig. 2C).

By comparing Pc structure with other proteins with a similar function and structure (endolysins, CHAP domains and others) by sequences alignment and superposition, we found that the catalytic residues are highly conserved (Supplementary Figure S4). In lysin LysGH15 (PDB ID: 4OLK)^24^ and LysK (PDB ID: 4CSH)^25^, the catalytic residues are Cys54, His117 and Glu134, while in the streptococcal phage lysin PlyC (PDB ID:4F88) the catalytic residues are Cys333 and His420^26^. The superposition found that the catalytic residues of Pc corresponds to Cys36, His99 and Asp116 (Fig. 2D, and Supplementary Figure S4B). These hypothetical catalytic residues are close to the hydrophobic cleft, which supports the possibility that the catalytic part of the molecule is located in the hydrophobic groove.

### Lytic spectrum of ClyF

**Figure 3:**
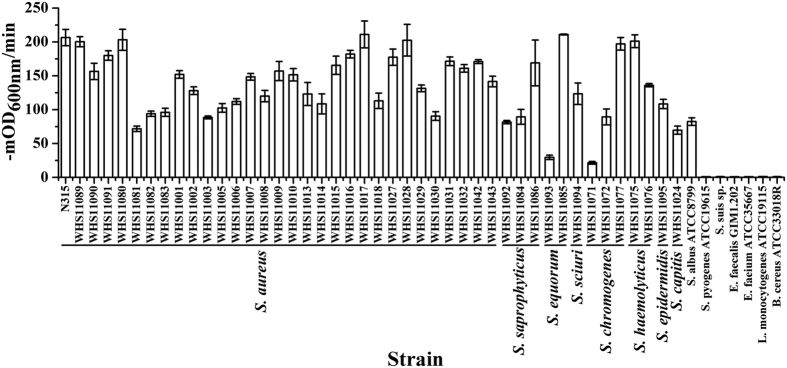
Lytic spectrum of ClyF. Multiple strains of staphylococci (including *S. aureus, S. saprophyticus, S. equorum, S. sciuri, S. chromogenes, S. haemolyticus, S. epidermidis, S. capitis*, and *S. albus*), streptococci, enterococci and *Listeria* were tested for susceptibility to ClyF. All the strains are washed once with PBS and treated with 25 μg/ml ClyF at 37 °C for 60 min. The final result is presented as rate of lysis (−mOD_600nm_/min) according to the decrease in OD_600nm_ for each strain in the first 3 min. Groups treated with PBS were used as controls, each assay was repeated for at least three times. Mean values are plotted, and error bars represent 1× standard deviation.

The susceptibility tests showed that ClyF was active against all staphylococcal isolates tested in PBS, including *S. aureus, S. saprophyticus, S. equorum, S. sciuri, S. chromogenes, S. haemolyticus, S. epidermidis, S. capitis*, and *S. albus*, but not other species tested (Fig. 3). Intra-species differences in initial rate of lysis were observed with the maximum rate of 211 ± 19.8 −mOD_600nm_/min versus the minimal rate of 71.7 ± 4.0 −mOD_600nm_/min in *S. aureus*. Similarly, a 7-fold difference was observed for two *S. equorum* isolates (29.6 ± 3.2 vs 210.9 ± 0.6 −mOD_600nm_/min for WHS11093 and WHS11085, respectively) and for two *S. chromogenes* isolates (21.5 ± 2.0 vs 197.2 ± 9.2 −mOD_600nm_/min for WHS11071 and WHS11077, respectively). However, the ultimate lysis outcomes in OD_600nm_ were close between these two *S. equorum* isolates (Supplementary Fig. S5). The observed difference in lytic rate may be due to the strain-to-strain variation of these clinical isolates, a phenomenon that was also observed by others^16^,^27^,^28^.

### Comparison of ClyF activity with other lysins

**Figure 4:**
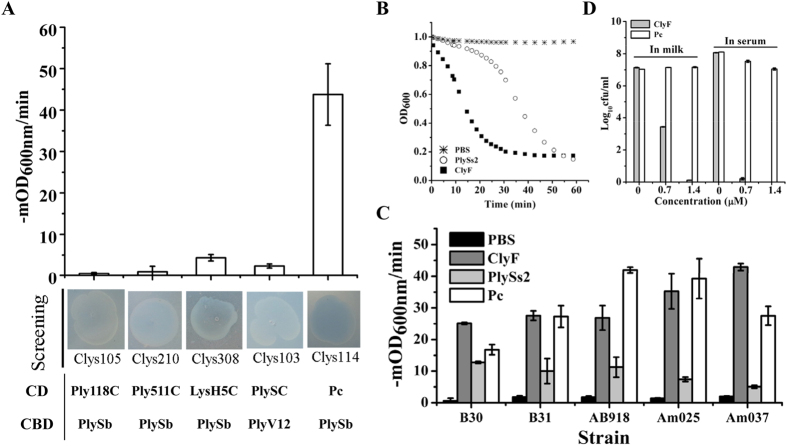
Comparison ClyF activity with other lysins. (**A**) Comparison the lytic rate (−mOD_600nm_/min) of ClyF against *S. aureus* N315 with that of four other chimeolysins discovered on the screening plates. (**B**) The changes of OD_600nm_ of *S. aureus* N315 exposed to an equal molar amount (0.35 μM) of ClyF and PlySs2. (**C**) Lytic rates of ClyF, PlySs2 and Pc (0.35 μM each) against several *S. aureus* clinical isolates. (**D**) Log killing abilities of ClyF and Pc against *S. aureus* N315 in milk and serum. *S. aureus* N315 resuspended in pasteurized milk and mouse serum were treated with different concentrations (0–1.4 μM) of ClyF or Pc at 37 °C for 1 h, respectively, the residual viable cell number was calculated by plating serial dilutions onto Baird-Parker agar plates. Each assay was repeated for at least three times. Mean values are plotted, and error bars represent 1× standard deviation.

Four additional chimeolysins, namely Clys103 (chimeolysin purified from clone AK103), Clys105 (chimeolysin purified from clone AK105), Clys210 (chimeolysin purified from clone AK210) and Clys308 (chimeolysin purified from clone AK308), were discovered on the screening plates (Supplementary Fig. S2C). Their antibacterial activities against *S. aureus* N315 were further examined in comparison with ClyF. Results showed that the activity of Clys114 (ClyF) against *S. aureus* N315 was much higher (10–87 folds faster) than that of these four chimeolysins, quite consistent with the observations on the screening plates (Fig. 4A). Next, we examined the activity of ClyF in comparison with one of its parental lysin PlySs2 (Supplementary Fig. S2D) in PBS buffer. Results showed that the lytic profile of ClyF against *S. aureus* N315 was quite different from that of PlySs2, ClyF caused a rapid decrease in turbidity, while a lag phase (about 15 min) occurred for PlySs2 although the final outcome of its lysis was close to that of ClyF (Fig. 4B). The tests against five other clinical *S. aureus* isolates further validated the improved lytic rate of ClyF, 2.0–8.5 folds faster than that of PlySs2 (Fig. 4C). Although Pc alone shows high staphylolytic activity in buffer^19^, we suspected that the PlySb CBD could enhance the staphylococcal-targeting ability of ClyF in bio-matrix conditions. To test this hypothesis, we compared the activities of ClyF and Pc in several conditions, including PBS buffer, pasteurized milk, mouse serum, and fresh pigskin surface. Results showed that there was no obvious difference in bacteriolytic activity between ClyF and Pc in PBS buffer (Fig. 4C). However, ClyF displayed a much higher bactericidal activity than Pc in milk, serum (Fig. 4D), and on pigskin (Supplementary Fig. S6). In addition, we also noted that the chimera ClyF showed a significantly (p < 0.01) higher activity than Pc under pH 8 to 10 (Supplementary Fig. S3).

### Biofilm removing efficacy of ClyF

**Figure 5:**
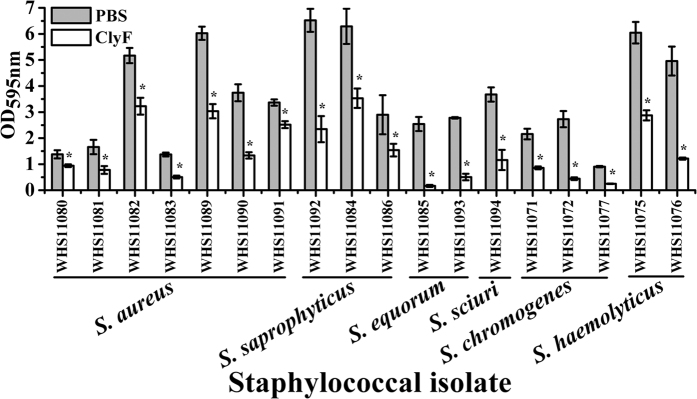
Biofilm removing efficacy of ClyF. The biofilm mass in each well was determined by crystal violet assay and compared with that of PBS treated wells. Data were analyzed by one-way ANOVA. Error bars represent standard deviation; * denotes p < 0.05.

The degradation efficiency of ClyF against established biofilms formed by various staphylococcal isolates were evaluated. The CV staining assay showed that all staphylococcal isolates tested could form biofilms and all biofilms showed good susceptibility to ClyF (Fig. 5). Indeed, significant decreases in biofilm mass from 25.2% (i.e. OD_595nm_ from 3.367 ± 0.122 to 2.519 ± 0.134 for *S. aureus* WHS11091) to 93.5% (i.e. OD_595nm_ from 2.542 ± 0.272 to 0.166 ± 0.039 for *S. equorum* WHS11085) were observed after ClyF treatment (Fig. 5).

### Efficacy of ClyF in a systemic infection model of MRSA bacteremia

**Figure 6:**
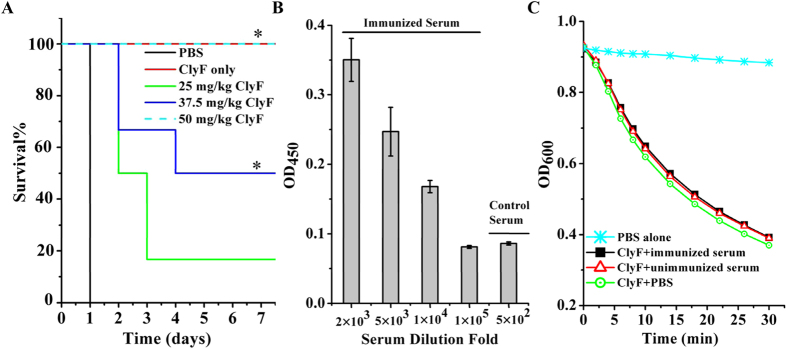
Efficacy of ClyF in a systemic mouse model of MRSA bacteremia. (**A**) Dose-dependent rescue curves of ClyF. Mice were intraperitoneally injected with 6 × 10^7^ CFU of MRSA WHS11081. Three hours post-injection, animals are divided into four groups randomly. Three groups (6 each) received 25, 37.5 and 50 mg/kg ClyF intraperitoneally, respectively, and the fourth group (n = 5) was injected with PBS buffer only (Blank line). Another group (n = 3) without MRSA infection was given 100 mg/kg ClyF only (Red line). The survival rates of all the groups were recorded over 7 days. Survival data of ClyF treated groups were analyzed by one-way ANOVA in comparison with that of the PBS treated control. * denotes p < 0.05. (**B**) Titers of anti-ClyF antibody in mice serum. The nonimmunized mouse serum was used as control. Error bars represent standard deviation. (**C**) Effect of ClyF-immunized serum on the lytic activity of ClyF *in vitro*. The nonimmunized mouse serum was used as negative control.

The *in vivo* antibacterial activity of ClyF was evaluated in a mouse model of MRSA bacteremia. As shown in Fig. 6, 100% of the mice challenged with 6 × 10^7^ CFU WHS11081/mouse and treated with PBS died within 24 h, while a dose-dependent rescue was observed in mice treated with ClyF. Indeed, 20% (1/6), 50% (3/6) and 100% (6/6) survival rates were observed in mice treated with 25, 37.5 and 50 mg/kg ClyF, respectively. Statistical analysis showed that the survival rates in groups treated with 37.5 and 50 mg/kg ClyF have significant differences (P < 0.05) in comparison with that of PBS treated controls (Fig. 6A). Further tests showed that no mice died after exposure to ClyF, either in a single intraperitoneal administration of a high dose (100 mg/kg, n = 3, Fig. 6A) or repeated intraperitoneal administration of a low dose (25 mg/kg/day for five continuous days, n = 3, data not shown). The antibody protection assay demonstrated that repeated injection of ClyF could induce immune responses in mice (Fig. 6B). However, the immunized serum showed no obvious neutralization effect on the lytic activity of ClyF *in vitro* (Fig. 6C).

### Efficacy of ClyF in a mouse model of burn wound infection

**Figure 7:**
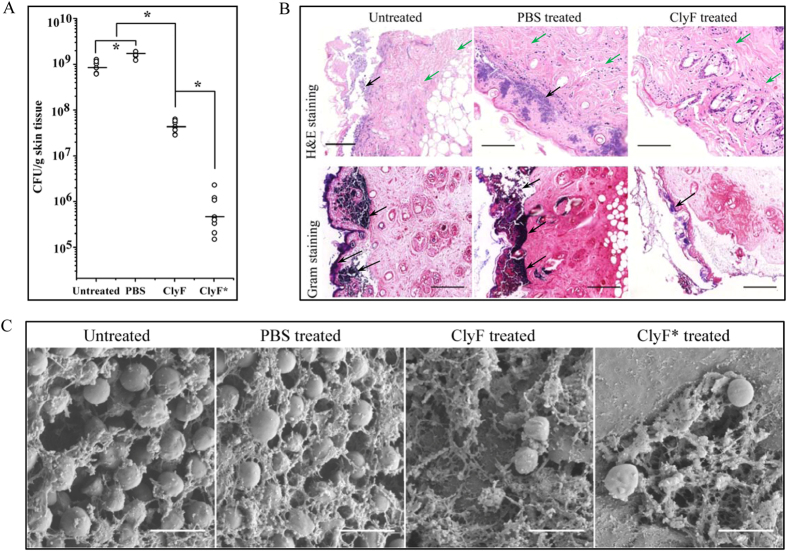
Efficacy of ClyF in a mouse model of burn wound infection. (**A**) Log killing efficacy of ClyF against *S. aureus* biofilms formed in burn wounds. *S. aureus* WHS11081 was inoculated onto the wounded dorsum skin of each mouse for 24 h to allow biofilm formation. After applied topically with ClyF for 24 h, the bioburden in each skin sample was homogenized and calculated by plating onto Baird-Parker agar plates. Untreated and PBS treated groups were used as controls. Data was analyzed by one-way ANOVA, each cycle represents an individual mouse, bars indicate mean, and * denotes p < 0.05. (**B**) H&E and Gram staining analysis of *S. aureus* biofilms in burn wounds. Tissue sections were imaged at x400 magnification by an automatic digital slide scanner. Bar: 100 μm. (**C**) SEM analysis of the *S. aureus* biofilms in burn wounds. Images were taken at x20,000 magnification under the same instrument conditions. ClyF* represents the group treated with 0.1 mg ClyF twice in a 6 h interval. Bar: 1 μm.

The biofilm degradation efficiency of ClyF against staphylococci *in vivo* was evaluated in a mouse model of burn wound infected by MRSA WHS11081 (1 × 10^7^ CFU/mouse). As shown in Fig. 7A, the average bioburden on burn wound skin surface was increased to 9.2 × 10^8^ cfu/g tissue (about 7.97 × 10^7^ cfu/mouse) in the untreated group. While the average bioburden was increased to 1.6 × 10^9^ cfu/g tissue (about 2.13 × 10^8^ cfu/mouse) in PBS treated group, and in groups treated with 0.1 mg ClyF in a single dose or twice (ClyF*), the average bioburden was reduced to 4.6 × 10^7^ cfu/g tissue (a reduction of 1.5 log10, about 5.34 × 10^6^ cfu/mouse) and 7.7 × 10^5^ cfu/g tissue (a reduction of 3.3 log10, about 8.47 × 10^4^ cfu/mouse), respectively. The histological analysis showed that dense *S. aureus* community (purple-blue) existed below the epidermis of the skin in the untreated and the PBS treated groups, while less *S. aureus* clump was observed in the ClyF treated group (Fig. 7B, top row). Moreover, obvious fibrous matrix material could be seen beneath the *S. aureus* biofilm tissues (green arrows), and the accumulation of immune cells was observed under the fibrous layer in all treatment groups (Supplementary Fig. S7). The Gram staining analysis also showed heavy *S. aureus* clump in the untreated and the PBS treated groups (blank arrows), and reduced clusters of *S. aureus* at the wound surface in the ClyF treated group (Fig. 7B, bottom row, blue).

SEM was performed to further characterize the development of *S. aureus* biofilms. As shown in Fig. 7C, the wound skin surfaces in the untreated and the PBS treated groups were covered by dense and thick biofilms, including distinct matrix fibers and amorphous encasing material. While, in the group treated with ClyF in a single dose or twice doses, the biofilms were observed to be degraded and destroyed in the wounds (Supplementary Fig. S8). These results were consistent with the observations in the section staining analysis.

## Discussion

The increased emergency of MDR pathogens worldwide awakens some old therapeutics (i.e. phage therapy^29^) and also, evokes lots of new strategies. One promising alternative among these new strategies is the lysin-derived therapy developed in the latest 15 years. Several key advantages of lysins include: 1) just killing a designated special or closely related species, without disturbing normal microbial flora; 2) robust killing efficacy against both planktonic and biofilm bacteria without evoking detectable resistance. Although there are no lysin-derived drugs on the market yet, the recent progresses in clinical trials for two staphylococcal lysins (i.e. CF-301^30^ and P128^31^) suggest bright potential for treating bacterial infections by lysins.

In the present study, we described the rapid screening of chimeolysins against staphylococci by using the induced lysis-based screening method. The key features of the screening method contain: 1) a novel chimeric enzyme, ClyN, that induces the autolysis of the host *E. coli* cells from within upon induction with IPTG; and 2) an *E. coli* expression library constructed by random shuffling of CDs and CBDs from different lysins under the control of another promoter (i.e. P_BAD_). Transferring plasmid pET-*clyN* into the expression library allows us to find the clones that expressing active bactericidal chimeolysins directly on the lawn of a target strain. By using of this screening method one chimeolysin, ClyF, was found to show the clearest zone on the *S. aureus* N315 plates. Further expression-based activity tests confirmed that ClyF has a much higher antibacterial activity than other lysins sharing the same CBD (Fig. 4A). These results demonstrated that the screening method is indeed a useful tool for rapid identification of active chimeolysins.

Sequencing analysis showed that ClyF consists of the CBD domain from lysin PlySs2 (PlySb) and the catalytic domain from lysin Ply187 (Pc). PlySs2, also referred to as CF-301, is a unique lysin derived from *S. suis* phage that shows high lytic activity against streptococci and staphylococci^28^. Our previous study showed that the CD of PlySs2 alone only has a very weak lytic activity, and the CBD of PlySs2 (PlySb) has high affinities for streptococci^32^ and some staphylococci (unpublished data), indicating that the broad binding ability of PlySb contributes significantly to the broad lytic spectrum of PlySs2. However, the host range of ClyF is found to be restricted to staphylococci (Fig. 3). This may be due to the hydrolase specificity of its CD donor Pc, which is derived from a staphylococci-targeted lysin. But as shown in the secondary (Fig. 1H and Table S3) and 3D structure (Fig. 2) analysis of ClyF, PlySb domain could endow the new chimera ClyF some new characteristics. First, the antiparallel-rich PlySb domain could enhance the flexibility of ClyF under thermal and different pH conditions. As revealed by the circular dichroism denaturation test and the pH-dependent lytic activity assay, the Tm of ClyF increases 9.3 °C (Fig. 1I) and the pH tolerance of ClyF is extended to pH 8–10 (Supplementary Fig. S3) compared to that of Pc. Second, the 3D structure modeling of Pc and ClyF showed that the PlySb domain contains many hydrophilic and charged residues (Fig. 2C), which could contribute also to improve the pH tolerance of ClyF in extreme acidic (pH 2–4) or alkaline (pH 8–10) environments.

The superposition analysis revealed that the catalytic residues of Pc is probably Cys36, His99 and Asp116 (Fig. 2D, and Supplementary Fig. S4B), which may be critical for its peptidoglycan hydrolase activity. The importance of Cys and His residues have been evidenced in some lysins that sharing a similar domain with Pc by site-directed mutation examination, for instance in PlyC^26^ and LysGH15^24^. The comparison analysis also showed that the Asp116 is not as highly conserved as Cys36 and His99 in Pc (Supplementary Figure S4A), however, Asp116 may be involved in the formation of a special catalytic triad in combination with the other two residues, similar as the catalytic triad formed in the mycobacteriophage lysin B^33^, the CHAP domain of the enzyme (PDB code 2K3A) from *Staphylococcus saprophyticus*^34^, and the *S. aureus* bacteriophage lysin LysK^25^. Although our analysis revealed some new insights into the catalytic mechanism of Pc, the quantified contribution of these residues still needs further study.

One outstanding property of ClyF distinguishing it from other lysins is its robust lytic activity both in buffer and bio-matrices (i.e. milk and serum). Reductions of 3.1 and 7 log10 in viable cell number were observed after treatment with 25 μg/ml ClyF in PBS for 5 min (Fig. 1A), and 40 μg/ml ClyF in milk for 1 h (Fig. 4D), respectively. Such a high lytic activity in milk was not observed for many other staphylococcal lysins. Considering the contaminations of staphylococci in cow mastitis and milk preservation, ClyF could also be exploited to prevent/remove staphylococci in milk conditions. Interestingly, an improved staphylolytic activity, killing > 7 logs after treatment with 20 μg/ml ClyF for 1 h (Fig. 4D), was observed in serum, suggesting that ClyF has good potential in controlling blood infections caused by *S. aureus*, for instance, bacteremia.

The *in vivo* studies also demonstrated that ClyF has good activities against *S. aureus* infections in mouse models. When applied systemically, ClyF showed a dose-dependent rescue efficacy in a mouse model of MRSA bacteremia (Fig. 6A). Although ClyF evoked the immune response, the antibodies seem not neutralizing the activity of ClyF, suggesting that either the anti-ClyF antibodies do not hinder the activity or they do not efficiently compete for the binding of ClyF to its substrate as explained by others^14^,^16^. When applying topically, ClyF showed good biofilm eradication efficacy against MRSA colonized in mice burn wounds (Fig. 7). Burn wounds infected with MRSA may progress into cellulitis and more serious invasive disease, e.g. septicaemia, in hospital^35^,^36^, and thus becomes a problem that difficult to deal with. Although applying ClyF once has showed significant reduction of the bacteria in the wound, dosing ClyF every 6 hours (twice) demonstrated much better antibacterial activity as shown in Fig. 7A. These results showed that repeated treatment may be needed when applying ClyF topically. However, the formulation and storing stability of ClyF still need further testing before clinical application.

Besides ClyF, several other chimeolysins against *S. aureus* have also been developed in recent years, including P128^31^, ClyS^16^, PRF-119^37^, Ply187AN-KSH3b^38^, ClyH^19^, and Lys170-87^39^. Each of them has been demonstrated effective against planktonic *S. aureus in vitro*, and in animal infection models. P128 has also showed good lytic activity against *S. aureus* in simulated nasal fluid^31^ and human blood^40^. Several chimeolysins also showed good degradation efficacy against *S. aureus* biofilms formed in microplates (i.e. ClyH and Ply187AN-KSH3b). However, as far as we know, ClyF is the first one that shows good bactericidal activity against both planktonic and biofilm *S. aureus in vitro* and *in vivo*, and all coagulase-negative staphylococci tested (Fig. 3), a property that has not been tested for other chimeolysins.

To summarize, we reported here a novel chimeolysin, ClyF, which was identified from the induced lysis-based screening method and showed robust bactericidal activity against planktonic and biofilm MRSA both *in vitro* and *in vivo*. Taking into account its good stability and high staphylolytic activity against all staphylococci species tested, ClyF could be a useful antimicrobial to combat MDR staphylococci.

## Methods

### Bacterial strains

All bacterial strains (Supplementary Table S1) used in this study were grown in trypticase soy broth (TSB) medium at 37 °C, except *Escherichia coli* that was cultured in Luria-Broth (LB) medium. A collection of clinical staphylococcal isolates with different antibiogram profiles were identified by PCR-DNA sequencing analysis combined with biochemistry test using a MicroStation system (Biolog, GEN III Omnilog Combo Plus System, USA). The PCR profiles of the *SCCmec* and enterotoxin gene types of these staphylococcal isolates were listed in Supplementary Figures S9 and S10, respectively.

### On-plate bactericidal chimeolysin screening

Our previous study has reported the screening of streptococci-targeted chimeolysins from an *E. coli* expression library containing combinations of seven CDs (from lysins Ply187, Ply118, Ply511, PlyGBS, PlySs2, and PlyC) and three CBDs (from lysins PlySs2, PlyV12 and LysAB2)^22^. Ply187 is a staphylococci-targeted lysin coded by *S. aureus* phage 187^41^. Ply118 and Ply511 are Listeria phage derived lysins^42^. PlyGBS^43^, PlySs2^28^ and PlyC^44^ are streptococcal phage derived lysins, and PlySs2 also shows an extended lytic activity against staphylococci. PlyV12 is an enterococcal phage lysin with effective kill activity against *Enterococcus faecalis* and *E. faecium*^27^. LysAB2 is an *Acinetobacter baumannii* phage lysin that shows extended antibacterial activity against *S. aureus*^45^. Because several domains were derived from staphylolytic lysins, the expression library was also tested against staphylococci. Briefly, individual clones from the *E. coli* expression library were inoculated into 1 ml fresh LB medium with 0.2% L-arabinose in 24-well plates. After overnight incubation at 37 °C and replica plating, wells were supplied with 0.5 mM isopropyl-β-D-thiogalactoside (IPTG, to initiate the expression of ClyN that can cause the lysis of host *E. coli* cell from within), and immediately, a 5 μl aliquot of these cultures was spotted on top of LB-agar (LA) plates overlaid with soft-agar containing *S. aureus* N315. Clear zones were checked at drop deposition sites. Finally, the corresponding clones which generated the clear zones were sequenced from the replica plates. The sequences of chimeolysins obtained were listed in the Supplementary Material.

### Construction of expression plasmids

To obtain a higher expression level, chimeolysins from clones AK103 (Clys103), AK105 (Clys105), AK114 (Clys114, or ClyF), AK210 (Clys210) and AK308 (Clys308) were sub-cloned into pET28a(+) plasmid, using the following primers (Supplementary Table S2): Clys103-F/Clys103-R for Clys103, Clys105-F/ClyR-R for Clys105, ClyF-F/ClyR-R for ClyF, Clys210-F/ClyR-R for Clys210, Clys308-F/ClyR-R for Clys308. Each construct was transformed into *E. coli* BL21(DE3) through the heat-shock method^46^, selected on LB agar plates containing 50 μg/ml Kanamycin, and confirmed by sequencing.

### Protein purification

Recombinant proteins were purified through Ni-NTA affinity chromatography as described previously with minor modifications^47^. Briefly, bacterial cells were induced with 0.2 mM IPTG overnight at 16 °C and collected for protein purification after sonication on ice. Purification was performed by washing and eluting with 60 and 250 mM imidazole, respectively. After dialyzed against PBS buffer (137 mM NaCl, 2.7 mM KCl, 4.3 mM Na_2_HPO_4_·H_2_O, 1.4 mM KH_2_PO_4_, pH 7.4), purified proteins were stored at −80 °C till use.

The parental lysins Pc (CD of the Ply187 lysin) and PlySs2 were purified through Ni-NTA affinity chromatography as described previously in our laboratory^19^,^32^, specifically, eluting with 265 mM and 400 mM imidazole, respectively, followed by dialyzing against PBS.

### Lytic activity assay

The bacteriolytic activity was determined as previously described^48^, with minor modifications. Briefly, bacteria cells were cultured overnight, centrifuged, and resuspended in PBS (pH 7.4) to a final OD_600nm_ of 0.8–1.0. After mixing 195 μl of bacterial suspension with 5 μl of lysin (final concentration of 25 μg/ml) in a 96-well plate (Perkin-Elmer, USA), the drop of OD_600nm_ in each well was monitored immediately by a microplate reader (Synergy H1, BioTek, USA) at 37 °C for 60 min. The bacterial suspension was mixed with ClyF by using of a multi-channel pipette in order to reduce the time lapse before reading (less than 5 s). The rate of lysis was measured as the drop in milli-OD_600nm_ per minute (−mOD_600nm_/min) in the first 3 min as described elsewhere^43^. Meanwhile, the decrease in viable cells corresponding to the loss of turbidity was also tested by plating the aliquots from the lytic assay at various time points (0, 2, 5, and 15 min) to TSB agar for counting of CFU. Ten-fold dilution was performed immediately (to decrease ClyF rapidly to very low concentrations that show minimum lytic activities) to avoid possible killing during plating. To determine the influence of NaCl, EDTA and pH on the lytic activity of ClyF, *S. aureus* N315 cells were suspended in phosphate buffer containing various concentrations of NaCl (ranging from 50 to 500 mM) or EDTA (ranging from 50 to 500 μM), or universal buffers with pH ranging from 2 to 12. The universal buffer was made by mixing equal parts of 20 mM boric acid and 20 mM phosphoric acid, followed by titration with sodium hydroxide as described before^49^. After adding 25 μg/ml ClyF to the bacterial suspensions, OD_600nm_ drops were monitored by the microplate reader at 37 °C for 60 min. The relative activities of ClyF in different NaCl, EDTA, and pH buffers were calculated by deducting the background signal of PBS treated wells and normalized by comparison with the maximum activity of ClyF in these buffers. To evaluate the effect of temperature, ClyF was stored at different temperatures ranging from 4 to 100 °C for 60 min and then cooled to room temperature (RT) for 10 min (prior to use in the assay) to determine the residual enzymatic activity. To determine the storage stability of ClyF, ClyF was stored at RT, 4 °C or −80 °C for different time periods (0–10 months), and the remaining activity against *S. aureus* N315 was measured at 37 °C (the frozen solutions were equilibrated to RT by placing the tubes at RT for 30 min before the assay) following the protocol described above. All experiments have been repeated for at least three times (or at least three microplate wells for each repeat), using PBS treated wells as controls.

### Compare the lytic activity of ClyF with other lysins

ClyF was further compared with four other chimeolysins discovered on the screening plates, namely Clys105, Clys210, Clys308, and Clys103 (Clys105, Clys210 and Clys308 were found to share the same CBD with ClyF, and Clys103 shares the same CD with PlySs2). A 5 μl aliquot of each protein (0.87 μM each) was mixed with 195 μl *S. aureus* N315 suspension followed by monitoring the decrease of OD_600nm_ at 37 °C for 60 min. To compare the activity of ClyF with its parental lysin PlySs2, the lytic profiles of ClyF and PlySs2 (0.35 μM each) against six *S. aureus* isolates in PBS buffer were determined.

### Compare the lytic activity of ClyF with Pc in bio-matrix conditions

In order to study the possible role of PlySb played in ClyF, we compared the lytic activity of ClyF with that of Pc under various bio-matrix conditions, including PBS buffer, milk, mouse serum and fresh pigskin surface. For testing the activity in milk and serum, staphylococcal isolates were resuspended in pasteurized milk and serum, respectively, and treated with different concentrations of ClyF or Pc (0, 0.7, or 1.4 μM) at 37 °C for 60 min. The number of residual viable cell after each treatment was determined by plating on LA plates. For testing the activity on pigskin, fresh pigskins (pre-cut into 2 × 2 cm^2^ square and treated with UV light for 30 min) were inoculated with 20 μl of *S. aureus* N315 cells (about 1 × 10^7^ cfu) and incubated at 37 °C for 1 h. After 20 μl of 0.25 mg/ml ClyF or Pc was applied for various times (1–4 h), pigskin was cut up and vortexed vigorously in 0.5 ml PBS for 1 min, then the viable cell number was calculated by plating serial dilutions on Baird-Parker agar plates (selective culture for *S. aureus*). All the above experiments were performed in triplicate using PBS treated groups as controls.

### Biofilm removing efficacy of ClyF

The efficiency of ClyF against established biofilms was determined by crystal violet (CV) staining^50^. Briefly, staphylococcal isolates, including *S. aureus, Staphylococcus saprophyticus, Staphylococcus equorum, Staphylococcus sciuri, Staphylococcus chromogenes* and *Staphylococcus haemolyticus*, were cultured in 96-well polystyrene plates (Tissue culture treated, Nest Biotech Co., China) supplied with TSBG (TSB with 1% glucose) medium for 24 h to develop biofilms. After washing twice with PBS, wells were treated either with 200 μl of 100 μg/ml ClyF, or an equal volume of PBS, at 37 °C for 45 min. After washing two times with PBS, wells were dried and stained with 200 μl of 0.1% CV (Merck, USA) at RT for 5 min. Following that, wells were washed two times with distilled water, solubilized with 200 μl ethanol and measured by a microplate reader at OD_595nm_ as described elsewhere^51^. The final biofilm masses in ClyF treated wells were analyzed by one-way analyses of variance (ANOVA) in comparison with that of the PBS treated wells. Difference with p < 0.05 was considered statistically significant. All experiments have been repeated for at least three times (at least triplicate microplate wells for each repeat).

### Immunological neutralization test

The neutralization effect of ClyF-specific antibodies on the activity of ClyF was tested as described previously^16^. Briefly, mice were injected with ClyF (0.5 mg) 3 times on 10-day intervals. Mice sera were sampled 15 days after the last injection, the titers of ClyF-specific antibody (>10,000) were determined by coating ClyF on a microplate and using horseradish peroxidase-conjugated goat anti-mouse IgG following the instructions of a commercial ELISA kit (QF-Bio, Shanghai, China). To determine the neutralization effect of immunized serum, ClyF (20 μg/ml in 90 μl volume) was pre-mixed with 10 μl of either ClyF immunized mouse serum (the final antibody titer was about 1:1,000), or preimmune serum, or PBS in a 96-well plate at 37 °C for 15 min. After adding an equal volume of *S. aureus* N315 bacterial suspension (100 μl to give a final ClyF concentration of 10 μg/ml), the decrease of OD_600nm_ in wells treated with the immunized serum were measured by a microplate reader and compared to those of preimmune serum and PBS treated wells.

### Ethics statement

All mouse experiments were carried out in an ABSL-2 lab and all experimental methods were carried out in accordance with the regulations and guidelines set forth by the Animal Experiments Committee of Wuhan Institute of Virology, Chinese Academy of Sciences. All experimental protocols were approved by the Animal Experiments Committee of Wuhan Institute of Virology, Chinese Academy of Sciences (approval no. WIVA17201401). Animals were cared in individually ventilated cages following a set of animal welfare and ethical criteria during the experiences, and euthanized at the end of observation.

### Systemic infection model of MRSA bacteremia

In the mouse systemic infection model, female BALB/c mice (SPF-level, 6–8 weeks old, obtained from Wuhan Institute of Biological Products Co., Ltd, Wuhan, China) were injected intraperitoneally with different concentrations of MRSA WHS11081 to determine the minimal lethal dose (MLD) that caused 100% mortality within one day. Then, mice were inoculated intraperitoneally with 2 × MLD of WHS11081 cells (6 × 10^7^ CFU/mouse in 200 μl volume). Three hours after challenging, mice were divided into 4 groups randomly. Three groups (n = 6) received 25, 37.5 and 50 mg/kg ClyF intra-peritoneally (in a volume of 0.5 ml), respectively, and the fourth group (n = 5) was injected with PBS buffer (0.5 ml) only. To evaluate the toxicity of ClyF, non-infected mice received a single-dose of 100 mg/kg ClyF (n = 3), or 25 mg/kg per day for five continuous days (n = 3). The survival rates for all groups were recorded over 7 days. Survival data of ClyF treated groups were analyzed by one-way ANOVA in comparison with that of the PBS treated control.

### Topical infection model of burn wound

In the burn wound skin model, 6–8 weeks old female BALB/c mice (SPF-level, and confirmed by plating on Baird Parker plates to exclude any natural staphylococcal flora on the mouse skin) were anesthetized by intraperitoneal injection of pentobarbital at 50 mg/kg and then shaved with an electric razor to create a 2-cm^2^ hairless area on dorsum as described^52^. Partial thickness burn was achieved by exposure of the naked skin to 80 °C water for 10 s. The burn was then inoculated with 1 × 10^7^ CFU WHS11081 (in 5 μl TSB). At 24 h post-colonization, mice were randomly separated into four groups as following: group 1 was left untreated (n = 6); group 2 was treated with 50 μl PBS only (n = 6); group 3 received a single-dose of 0.1 mg ClyF topically in 50 μl PBS (n = 8) and group 4 received two doses of 0.1 mg ClyF topically in 50 μl PBS at 6 h intervals (n = 8). 24 h post-treatment, mice were sacrificed by cervical dislocation method for skin harvesting. Specifically, all the infected wound area was excised, weighed, and homogenized in 1 ml PBS for 30 s. The viable cell number in each tissue sample was calculated by plating tissue dilutions onto Baird-Parker agar plates and analyzed by one-way ANOVA. To test whether ClyF carryover could have occurred during dilution and plating procedures, residual enzymatic activity of ClyF on the skin surface was tested. Briefly, tissue samples were homogenized in PBS and centrifuged at 10,000 g for 1 min, then the supernatant (100 μl) was mixed with *S. aureus* N315 cells and the enzymatic activity was determined immediately by monitoring the changes in OD_600nm_, using PBS as control. No decrease in OD_600nm_ was observed as monitored by the microplate reader, indicating that no active ClyF was remained after 24 h on the skin surface (data not shown).

### Hematoxylin and eosin staining

After 24 h of treatment, skin tissues taken for histological analysis were fixed with formalin and embedded in paraffin. Sections (Thermo Cytospin 4) were either Gram stained or stained with hematoxylin and eosin (H&E) using an automatic immunohistochemical stainer (Autostainer 480&PT, Thermo Scientific, USA). Samples were imaged and analyzed by an automatic digital slide scanner (Pannoramic MIDI, 3DHISTCH, Hungary).

### Scanning electron microscope

To visualize MRSA biofilms in burn wounds, skin tissues (treated with PBS or ClyF) were harvested, fixed with 2.5% glutaraldehyde and dehydrated by granted ethanol (from 30% to 100%). After treatment with critical point drying and sputter-coated with gold, tissue samples were examined by a scanning electron microscope (SEM, SU8010, Hitachi, Japan). Images were taken at ×20,000 magnification under the same instrument conditions.

### Circular dichroism

The circular dichroism spectra were collected with an Applied Photophysics Chirascan plus circular dichroism spectrometer (Leatherhead, UK). For native structure measurement, spectra of ClyF (7.7 μM) and Pc (10.7 μM) in 4 mM Tris-HCl (pH 7.4) were recorded from 190–260 nm (0.1 cm path length) at RT. The spectra of air and buffer were recorded as background and baseline, respectively. For evaluation of thermal stability, the melting temperature (Tm) of ClyF (38.5 μM) and Pc (21.4 μM) in 4 mM Tris-HCl (pH 7.4) were measured at 220 nm by recording the circular dichroism signal at a temperature range of 25–90 °C. The secondary structures and Tms of ClyF and Pc were calculated by CDNN V2.1 software supplied by the instrument provider.

### Construction of 3D models of Pc and ClyF

The 3D models of Pc and ClyF were constructed by Rosetta 3.5^23^. Rosetta provided both ab initio and comparative models of protein domains using the Rosetta fragment insertion method. Domains without a detectable PDB homolog were modeled with the Rosetta de novo protocol. Comparative models were built from parent PDBs detected by PSI-BLAST or HHSEARCH and aligned by HHSEARCH and SPARKS. Loop regions were assembled from fragments and optimized to fit the aligned template structure^53^. The manual model building was performed with Coot^54^. And the quality of modeled 3D structures was checked using MolProbity^55^.

## Additional Information

**How to cite this article**: Yang, H. *et al*. A novel chimeric lysin with robust antibacterial activity against planktonic and biofilm methicillin-resistant *Staphylococcus aureus. Sci. Rep.* **7**, 40182; doi: 10.1038/srep40182 (2017).

**Publisher's note:** Springer Nature remains neutral with regard to jurisdictional claims in published maps and institutional affiliations.

## Supplementary Material

Supplemental Material
