# Supplementary material for: A novel chimeric lysin with robust antibacterial activity against planktonic and biofilm methicillin-resistant Staphylococcus aureus
Source: Sci Rep. 2017 Jan 9;7:40182. doi: 10.1038/srep40182 (PMC5220359; doi:10.1038/srep40182)
Supplement: Supplemental Material [file srep40182-s1.pdf]

Supplementary material

A novel chimeric lysin with robust antibacterial activity against planktonic and biofilm methicillin-resistant *Staphylococcus aureus*

Hang Yang, Huaidong Zhang, Jing Wang, Junping Yu, Hongping Wei*

Key Laboratory of Special Pathogens and Biosafety, Center for Emerging Infectious Diseases, Wuhan Institute of Virology, Chinese Academy of Sciences, Wuhan 430071, China.

Sequences of chimeolysins tested:

Clys103 from clone AK103:

atgacaacagtaaatgaagcattaaataatgtacgcgctcaggttgggtccggtgtgtctgttggcaacggcgaatgctacgctttggct
agttggtacgagcgcatgattagtccggatgcaactgtcggacttggcgctggtgtgggctgggtcagcggtgcaatcggcgatacaa
tctctgccaaaaacatcggctcatcatacaactggcaagctaacggctggacagtttccacatctggtccatttaaagcaggtcagattgt
gacgcttggggcaacaccaggaaacccttacggacatgtggtaatcgtcgaagcagtggacggcgatcgcttgactattttggagcaa
aactacggcgggaaacgttatccggtccgtaattattacagcgctgcaagctatcgtcaacaggtcgtgcattacatcacatctagatta
aacggtggaagcactcctccaaaaccaaacactaagaaagtaaaagtacttaaacacgctaccaactggtctccatcaagtaaaggtg
caaaaatggcaagttttgtcaaaggcggtacatttgaagttaaacaacaacgaccaatttcttattcttactctaatcaagaatacttgattgt
aaataaaggaacagtacttggatgggttctttcccaagacattgaaggtggatatggttcagatagagttgggggaagcaaacctaaac
tacctgccggatttacgaaggaagaagctacgtttattaatggtaatgctcctattactactcgtaagaataaaccaagcttatcttctcaaa
cagctacaccattgtatccgggacaatctgtaagataccttggttggaaatctgctgagggatacatctggatttatgcaacagatggac
gttacatccctgtacgacctgtaggtaaggaagcatggggtacatttaagtaa;

Clys105 from clone AK105:

atgacctcatactactattctcgctccctggctaatgtgaacaaactggctgataacacgaaagctgctgcccgtaaactgctggattggt
cagaatcgaacggcattgaagtcctgatctatgaaaccattcgtacgaaagaacagcaagcggccaacgtgaatagtggcgcatccc
agaccatgcgcagctatcatctggtcggtcaagcactggattttgtgatggctaaaggcaaaaccgttgattggggtgcgtaccgcagc
gacaaaggtaaaaaattcgtggcaaaagctaaaagtctgggcttcgaatggggcggtgattggtccggttttgttgacaacccgcatct
gcaattcaattataaaggctacggcaccgatacgtttggcaaaggtgcaagcacctctaacagctctaaaccgtcagctgacaccaac
accaacagcctgggcctggtcgattatatgaacctgaataaactggacagttccttcgcgaatcgtaaaaaactggccaccagctatgg
catcaaaaactactctggcaccgcgacgcagaataccacgctgctggcgaaactgaaagccggtaaatctagatctcgttcctatcgc
gagacgggcactatgactgtcacggtcgatgctctcaatgttcgccgcgcgccaaatacttcaggcgagattgtagcagtatacaagc
gtggtgaatcatttgactatgatactgtcatcatcgatgtcaatggctatgtctgggtgtcttacattggcggcagcggcaaacgtaactac

gttgcgacgggcgctaccaaagacggtaagcgtttcggcaatgcttggggtacatttaaataa;

Clys114 (ClyF) from clone AK114:

atggcactgcctaaaacgggtaaaccaacggcaaaacaggtggttgactgggcaatcaatttaatcggcagtggtgtcgatgttgatg
gttattatggtcggcaatgttgggatttacctaactatatttttaatagatactggaactttaagacaccaggcaacgcaagagatatggcat
ggtatagatatcctgaagggtttaaagtgtttagaaacacttctgattttgtccctaaaccaggtgatatagcagtgtggacaggtggtaat
tacaattggaacacttggggacacactggtattgttgtaggtccatcaactaaaagttacttttatagtgtagatcagaattggaataactct
aactcttacgttggtagtcctgcagcaaagataaaacatagttattttggtgtaactcattttgttagacccgcatacaaagcagaaccgaa
acctacaccaccatctagatctcgttcctatcgcgagacgggcactatgactgtcacggtcgatgctctcaatgttcgccgcgcgccaa
atacttcaggcgagattgtagcagtatacaagcgtggtgaatcatttgactatgatactgtcatcatcgatgtcaatggctatgtctgggtg
tcttacattggcggcagcggcaaacgtaactacgttgcgacgggcgctaccaaagacggtaagcgtttcggcaatgcttggggtacat
ttaaataa;

Clys210 from clone AK210:

atggtcaaatacacggtggaaaataaaatcatcgctggtctgccgaaaggtaaactgaaaggcgcaaacttcgtcatcgcacacgaaa
ccgcgaactcaaaatcgacgattgataatgaagtgtcttatatgacccgtaactggaaaaatgcttttgttacgcatttcgtcggcggtgg
cggtcgcgtggttcaggttgcgaacgtcaattatgtgtcgtggggtgcgggccagtacgccaacagctattcttacgctcaagtggaac
tgtgccgtaccagcaatgcgaccacgttcaaaaaagattacgaagtgtactgtcagctgctggttgacctggcgaaaaaagccggtatt
ccgatcaccctggatagtggttccaaaacgtcagacaaaggcattaaatcgcataaatgggttgccgataaactgggcggcaccacg
caccaggacccgtatgcatacctgagctcttggggtatcagcaaagcacaattcgcttctgatctggccaaagttagtggcggtggcaa
caccggcacggcaccggcaaaaccgtctaccccggcaccgaaaccgagcaccccgtctacgaatctgtctagatctcgttcctatcg
cgagacgggcactatgactgtcacggtcgatgctctcaatgttcgccgcgcgccaaatacttcaggcgagattgtagcagtatacaag
cgtggtgaatcatttgactatgatactgtcatcatcgatgtcaatggctatgtctgggtgtcttacattggcggcagcggcaaacgtaacta
cgttgcgacgggcgctaccaaagacggtaagcgtttcggcaatgcttggggtacatttaaataa;

Clys308 from clone AK308:

atggaagtcgcaacgatgcaggcaaaactgaccaaaaaagaatttattgaatggctgaaaacctcggaaggcaaacagtacaacgca
gatggctggtatggttttcagtgcttcgactacgcgaacgccggttggaaagcactgtttggcctgctgctgaaaggtgttggcgccaa
agatattccgtttgcaaacaatttcgacggtctggcaaccgtctatcagaacacgccggattttctggctcaaccgggtgacatggtggtt
ttcggcagcaattatggtgccggctacggtcatgtggcttgggttatcgaagcgaccctggattatattatcgtgtacgaacagaactgg
ctgggcggtggctggaccgacggtgtgcagcaaccgggctctggttgggaaaaagttacgcgtcgccagcacgcgtatgattttccg
atgtggttcattcgtccgaattttaaaagtgaaaccgctccgcgcagtgtgcagtccccgacccaggcgagcaaaaaagaaacggcg
aaaccgcagccgaaagccgttgaactgaaaatcatcaaagatgtcgtgaaaggttacgacctgccgaaacgtggcagcaacccgaa
tttcattgtgatccataacgatgccggctctaaaggtgcaaccgctgaagcgtatcgtaacggtctggttaatgcaccgctgtcccgcct
ggaagctggtattgcgcattcatacgtctcgggcaataccgtgtggcaggcgctggatgaaagtcaagtgggttggcacacggccaa
ccagattggcaataaatatggctacggtatcgaagtttgccaaagcatgggtgccgacaacgcaacctttctgaaaaatgaacaggca
acgttccaagaatgtgcttctagatctcgttcctatcgcgagacgggcactatgactgtcacggtcgatgctctcaatgttcgccgcgcg
ccaaatacttcaggcgagattgtagcagtatacaagcgtggtgaatcatttgactatgatactgtcatcatcgatgtcaatggctatgtctg
ggtgtcttacattggcggcagcggcaaacgtaactacgttgcgacgggcgctaccaaagacggtaagcgtttcggcaatgcttggggt
acatttaaataa;

Supplementary Table S1 Strains used in this study.

Organism	Strain	Antibiogram[c]	Enterotoxin[d]	*SCCmec*[e]	Source[f]
Staphylococci					
S. aureus	WHS11089[a]	Kan^R, Van^I, Amp^R, Oxa^R	D, G, I	ND[g]	1
	WHS11090[a]	Kan^R, Van^S, Amp^R, Oxa^R	C, G, I	ND	1
	WHS11091[a]	Kan^R, Van^S, Amp^R, Oxa^R	D, G, I	ND	1
	WHS11080	Kan^S, Van^I, Amp^R, Oxa^S	D, J	ND	1
	WHS11081[a]	Kan^S, Van^I, Amp^R, Oxa^R	D, J	ND	1
	WHS11082[a]	Kan^S, Van^I, Amp^R, Oxa^R	D, J	ND	1
	WHS11083	Kan^S, Van^I, Amp^R, Oxa^S	D, J	ND	1
	WHS11001[a]	Amp^R, Oxa^R	ND	I	2
	WHS11002[a]	Amp^R, Oxa^R	ND	I	3
	WHS11003[a]	Amp^R, Oxa^R	ND	I	3
	WHS11005[a]	Amp^R, Oxa^R	ND	I	3
	WHS11006[a]	Amp^R, Oxa^R	ND	I	3
	WHS11007[a]	Amp^R, Oxa^R	ND	I	4
	WHS11008[a]	Amp^R, Oxa^R	ND	I	2
	WHS11009[a]	Amp^R, Oxa^R	ND	I	2
	WHS11010[a]	Amp^R, Oxa^R	ND	I	2
	WHS11013[a]	Amp^R, Oxa^R	ND	II	2
	WHS11014[a]	Amp^R, Oxa^R	ND	II	2
	WHS11015[a]	Amp^R, Oxa^R	ND	II	2
	WHS11016[a]	Amp^R, Oxa^R	ND	II	3
	WHS11017[a]	Amp^R, Oxa^R	ND	II	3
	WHS11018[a]	Amp^R, Oxa^R	ND	II	3
	WHS11027[a]	Amp^R, Oxa^R	ND	III	3
	WHS11028[a]	Amp^R, Oxa^R	ND	III	3
	WHS11029[a]	Amp^R, Oxa^R	ND	III	3
	WHS11030[a]	Amp^R, Oxa^R	ND	III	4
	WHS11031[a]	Amp^R, Oxa^R	ND	IV	4
	WHS11032[a]	Amp^R, Oxa^R	ND	IV	4
	WHS11042[a]	Amp^R, Oxa^R	ND	V	2
	WHS11043[a]	Amp^R, Oxa^R	ND	V	2
	N315[a]	Oxa^R	ND	II	5
	AB918[b]	Oxa^S	ND	ND	6
	B30	Oxa^S	ND	ND	4
	B31[a]	Oxa^R	ND	ND	7
	AM025[a]	Oxa^R	ND	III	7
	AM037[a]	Oxa^R	ND	IV	7
S. saprophyticus	WHS11092	Kan^S, Van^I, Amp^R, Oxa^S	G, I, J	ND	1
	WHS11084	Kan^S, Van^I, Amp^R, Oxa^S	I	ND	1
	WHS11086	Kan^S, Van^I, Amp^R, Oxa^R	D, G, I, J	ND	1
S. equorum	WHS11093	Kan^S, Van^S, Amp^R, Oxa^S	G, I	ND	1

	WHS11085	Kan^S, Van^I, Amp^R, Oxa^R	G, I	ND	1
S. sciuri	WHS11094	Kan^S, Van^I, Amp^R, Oxa^S	G, I	ND	1
S. chromogenes	WHS11071	Kan^S, Van^I, Amp^R, Oxa^S	D	ND	1
	WHS11072	Kan^S, Van^S, Amp^R, Oxa^S	D, I, J	ND	1
	WHS11077	Kan^S, Van^S, Amp^R, Oxa^S	J	ND	1
S. haemolyticus	WHS11075	Kan^S, Van^R, Amp^R, Oxa^S	D, I, J	ND	1
	WHS11076	Kan^S, Van^R, Amp^R, Oxa^S	G, I	ND	1
S. epidermidis	WHS11095	ND	ND	ND	1
S. capitis	WHS11024	ND	ND	ND	1
S. albus	ATCC 8799	ND	ND	ND	8
Other strains					
S. pyogenes	ATCC 19615	ND	ND	ND	9
S. suis	sp.	ND	ND	ND	3
E. faecalis	GIM1.202	ND	ND	ND	8
E. faecium	ATCC 35667	ND	ND	ND	8
L. monocytogenes	ATCC 19115	ND	ND	ND	8
B. cereus	ATCC 33018R	ND	ND	ND	3
E. coli	BL21(DE3)	Kan^S, Van^S, Amp^S, Oxa^S	ND	ND	3
E. coli	BL21(DE3)/pET-Pc	Kan^R, Van^S, Amp^S, Oxa^S	ND	ND	3
E. coli	BL21(DE3)/pET-PlySs2	Kan^R, Van^S, Amp^S, Oxa^S	ND	ND	3

[a] Methicillin-resistant Staphylococcus aureus, determined by PCR according to the method described [1];

[b] AB918 is short for CCTCC AB91118;

[c] Antibiogram: Kan, kanamycin; Van, vancomycin; Amp, ampicillin; Oxa, oxacillin; R, resistant; I, intermediate resistant; S, susceptible.

[d] The type of enterotoxin was determined by PCR according to the method described [2,3].

[e] The *SCCmec* type were determined by multiple-PCR according to the method described [4].

[f] Source: 1. Gift from Huanggang agricultural scientific academy, Huanggang, Hubei, China. 2. Isolated from Zhongnan Hospital of Wuhan University; 3. Laboratory collection; 4. Isolated from Wuhan Medical Treatment Center; 5. Provided by professor Xiancai Rao at Third Military Medical University, Chongqing, China; 6. Purchased from China Center For Type Culture Collection, Wuhan, China; 7. Isolated from Hubei Maternal and Child Health Hospital; 8. Purchased from Guangdong Culture Collection Center, China. 9. Purchased from Shanghai Shifeng Biological Technology Co., LTD, Shanghai, China.

[g] ND, not detected.

Supplementary Table S2 Primers used in this study.

Primers	Sequence (5’-3’)
Clys103-F	TATACCATGGGCATGACAACAGTAAATGAAG
Clys103-R	AATTCTCGAGCTTAAATGTACCCCATGC
Clys105-F	TATACCATGGGCATGACCTCATACTACTATTC
ClyF-F	TTAACCATGGGCATGGCACTGCCTAAAACG
Clys210-F	TATACCATGGGCATGGTCAAATACACGGTG
Clys308-F	TATACCATGGGCATGGAAGTCGCAACG
ClyR-R	ATATCTCGAGTTTGAAGGTACCCCATGCGTTG

Supplementary Table S3 Secondary structure of ClyF and its parental lysin Pc [a].

Secondary Structure	ClyF[b] (%)	Pc[b] (%)
Helix	8.2	17.7
Antiparallel	46.5	22.3
Parallel	4.1	5.4
Beta-Turn	16.8	18.9
Rndm. Coil	28.8	33.7

[a] CDNN V2.1 software is used to evaluate the secondary structure.

[b] ClyF (7.7 µM) and Pc (10.7 µM) are dissolved in 4 mM Tris-HCl, pH=7.4.

Supplementary Figure S1 Screening bactericidal chimeolysins against staphylococci.

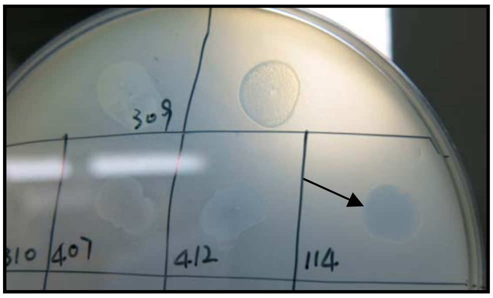

Supplementary Figure S1 Screening bactericidal chimeolysins against staphylococci. Clones from *E. coli* expression library are cultured with 0.2% L-arabinose overnight, and screened for generating clear zones on the soft agar plates overlaid with N315, as indicated by arrow.

Supplementary Figure S2 SDS-PAGE analysis of recombinant proteins.

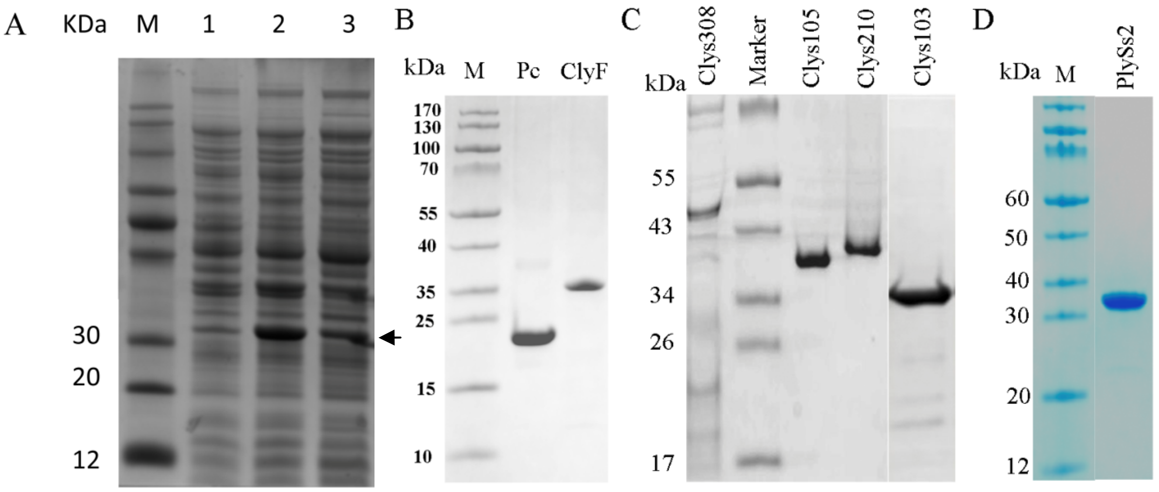

Supplementary Figure S2 SDS-PAGE analysis of recombinant proteins. (A) Induced expression of ClyF on 12% SDS-PAGE gels. Lanes: M: marker; 1: bacterial suspension before induction; 2: bacterial suspension after induction; 3: bacterial supernatant after induction. (B-D) Purified proteins analyzed on 12% SDS-PAGE gels.

Supplementary Figure S3 Comparison of the activity of ClyF with that of Pc under different pHs.

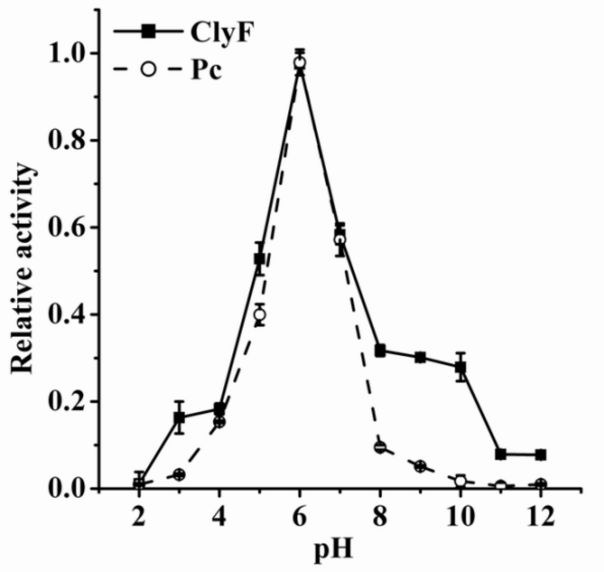

Supplementary Figure S3 Comparison of the activity of ClyF with that of Pc under different pHs.

Supplementary Figure S4 Sequence alignment and Superposition of Pc, LysK_CHAPK, LysGH15_CHAP and PlyC_CHAP.

A

B

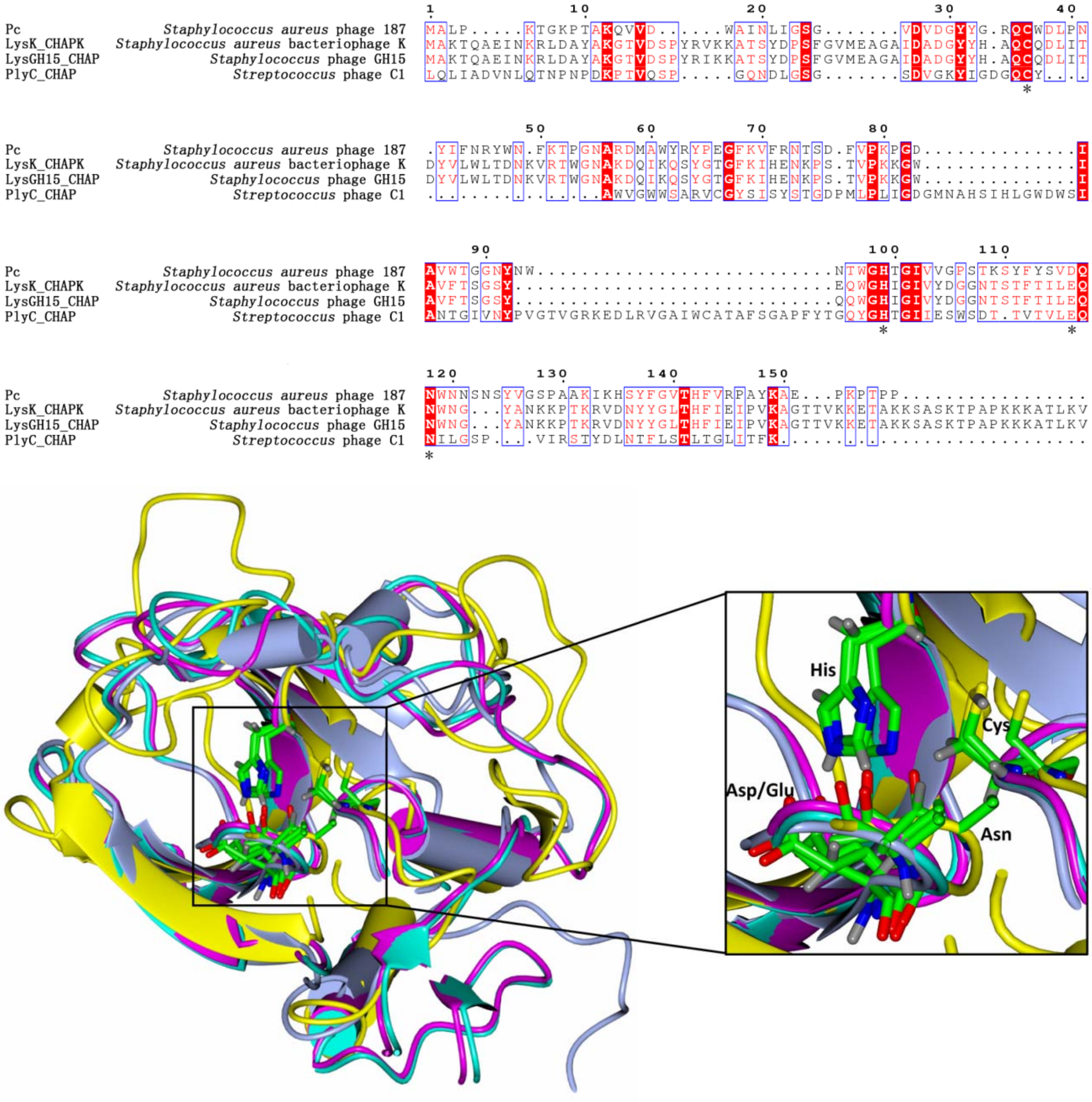

Supplementary Figure S4 Sequence alignment and Superposition of Pc, LysK_CHAPK, LysGH15_CHAP and PlyC_CHAP. (A) Sequence alignment analysis. The sequence of Pc is aligned with the CHAPK domain of LysK from *Staphylococcus aureus* bacteriophage K, the CHAP domain of LysGH15 from Staphylococcus phage GH15 and the CHAP domain of PlyC from Streptococcus phage C1. The conserved residues are colored in red, and the residues involved in catalysis are marked by *. (B) Superposition analysis in worms/tubes diagram. The worms/tubes diagram of Pc (colored in ice blue) is

superposed with that of LysK_CHAPK (colored in cyan), LysGH15_CHAP (colored in magenta) and PlyC_CHAP (colored in yellow). The boxed region shows the conserved residues in active sites.

Supplementary Figure S5 The lytic curves of ClyF against WHS11093 and WHS11085.

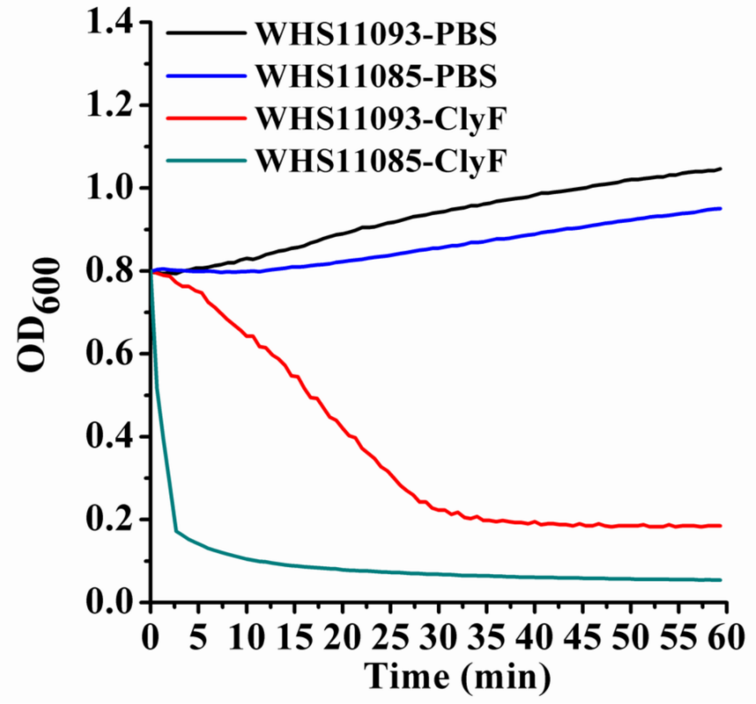

Supplementary Figure S5 The lytic curves of ClyF against WHS11093 and WHS11085. Cells are washed once with PBS and treated with 25 μg/ml ClyF at 37°C for 60 min. The changes of OD_{600} are monitored by a microplate reader.

Supplementary Figure S6 The efficacy of ClyF and Pc against *S. aureus* on pigskin surface.

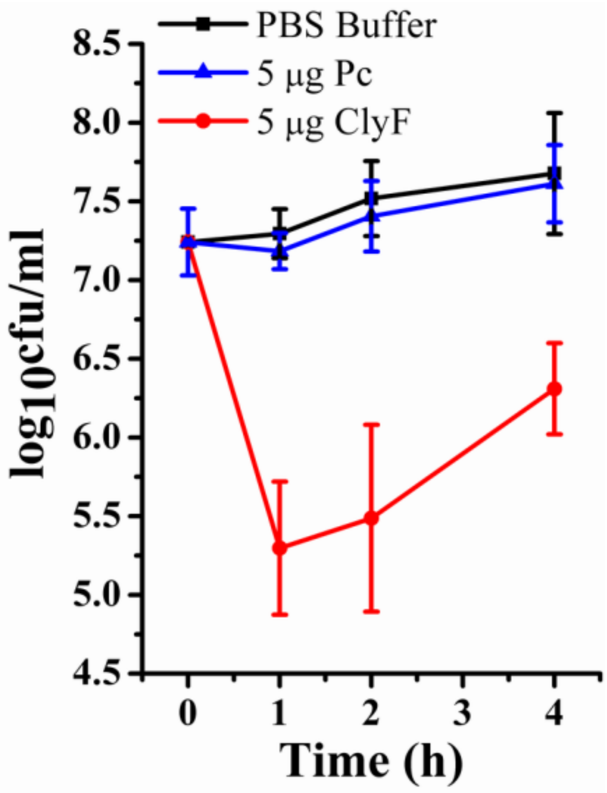

Supplementary Figure S6 The efficacy of ClyF and Pc against *S. aureus* on pigskin surface. N315 cells were inoculated on fresh pigskin surface (pre-cut into 2×2 cm^2 square and treated with UV for 30 min) and kept at RT for 1 h. Then the skin was treated with 20 µl of 0.25 mg/ml ClyF or Pc. The residual viable cell number on each pigskin is calculated at various times (1-4 h) by plating on Baird-Parker agar plates (*S. aureus* selective culture). The PBS treated groups are used as controls.

Supplementary Figure S7 H&E staining analysis of *S. aureus* biofilms.

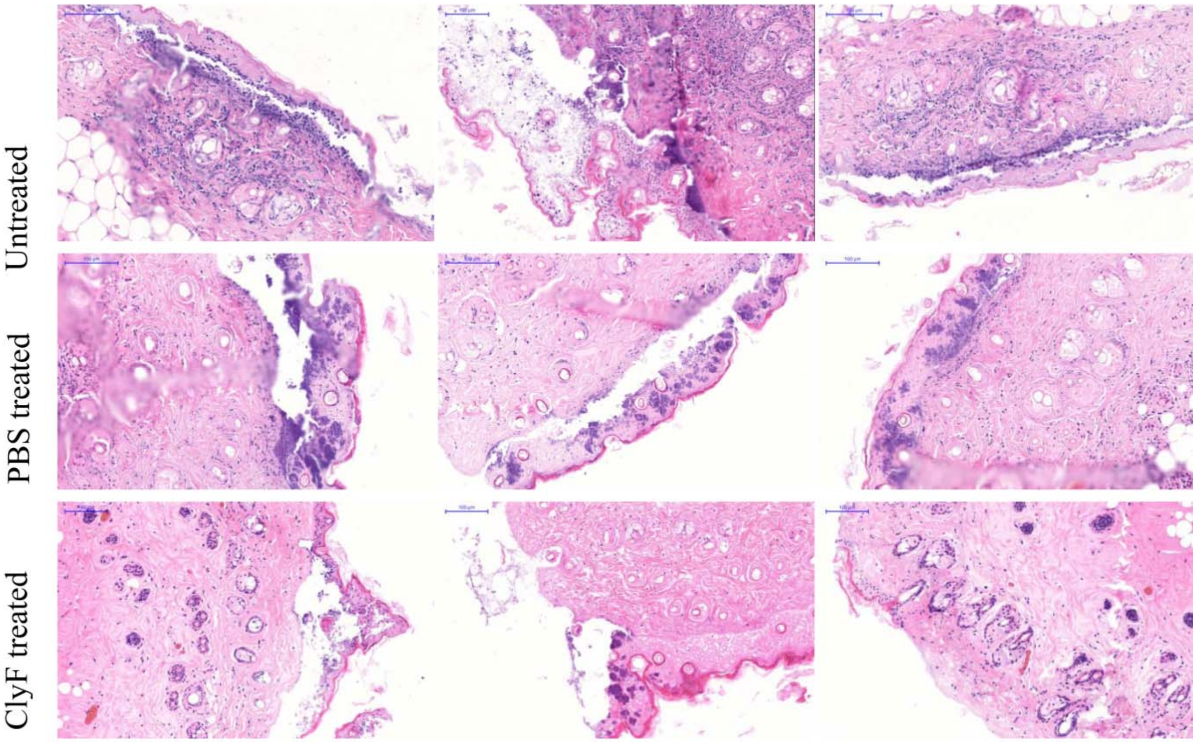

Supplementary Figure S7 H&E staining analysis of *S. aureus* biofilms. Tissue sections were imaged at ×400 magnification by an automatic digital slide scanner. Bar: 100 µm.

Supplementary Figure S8 SEM images of the *S. aureus* biofilms in burn wounds.

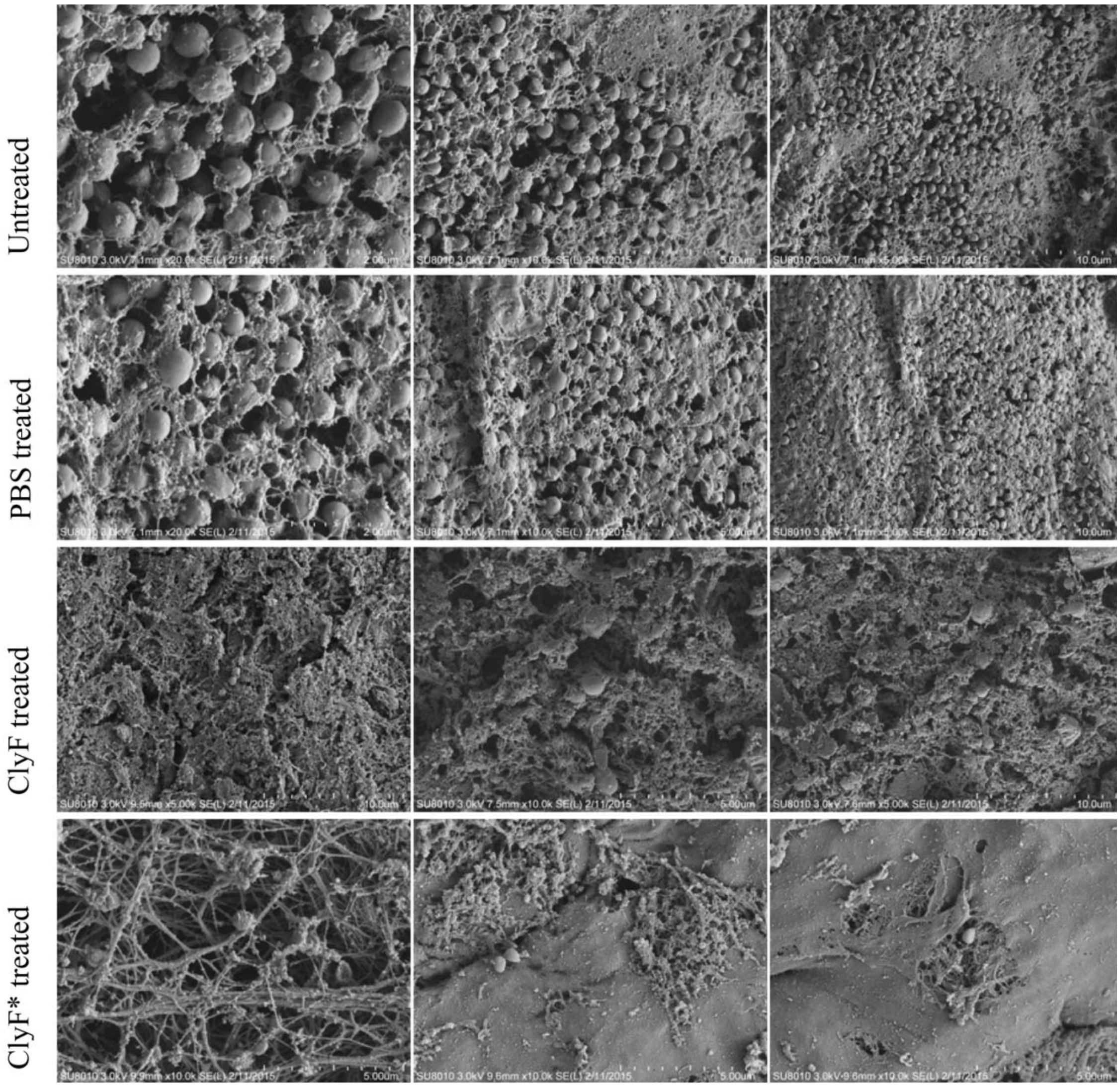

Supplementary Figure S8 SEM images of the *S. aureus* biofilms in burn wounds. ClyF* represents the group treated with 0.1 mg ClyF twice in a 6 h interval.

Supplementary Figure S9 The profiles of the *SCCmec* and enterotoxin gene types of staphylococcal isolates used in this study.

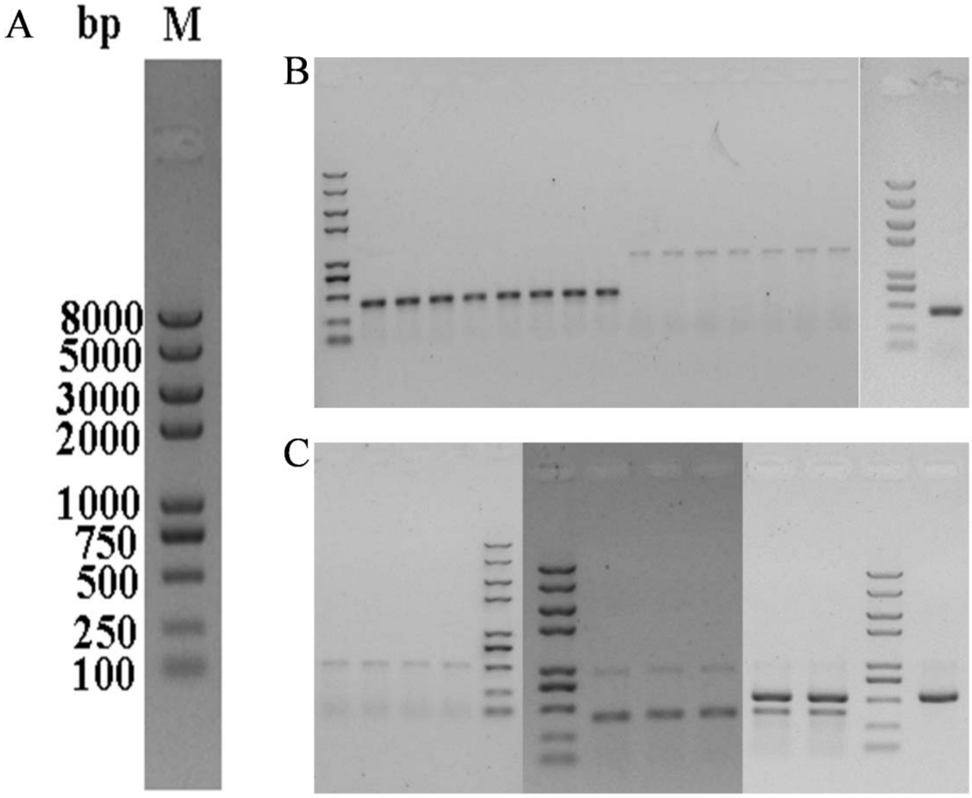

Supplementary Figure S9 The profiles of the *SCCmec* type and enterotoxin gene of staphylococcal isolates used in this study. (A) Standard DNA marker used in the assay. (B-C) Multiple-PCR identification of the *SCCmec* type of MRSA. (B) Lanes from left to right: Marker, WHS11001, WHS11002, WHS11003, WHS11005, WHS11006, WHS11007, WHS11008, WHS11009, WHS11013, WHS11014, WHS11015, WHS11016, WHS11017, WHS11018, N315, Marker, WHS11010; (C) Lanes from left to right: WHS11027, WHS11028, WHS11029, WHS11030, Marker, Marker, WHS11031, WHS11032, AM037, WHS11042, WHS11043, Marker, AM025.

Supplementary Figure S10 The profiles of the enterotoxin gene types of staphylococcal isolates used in this study.

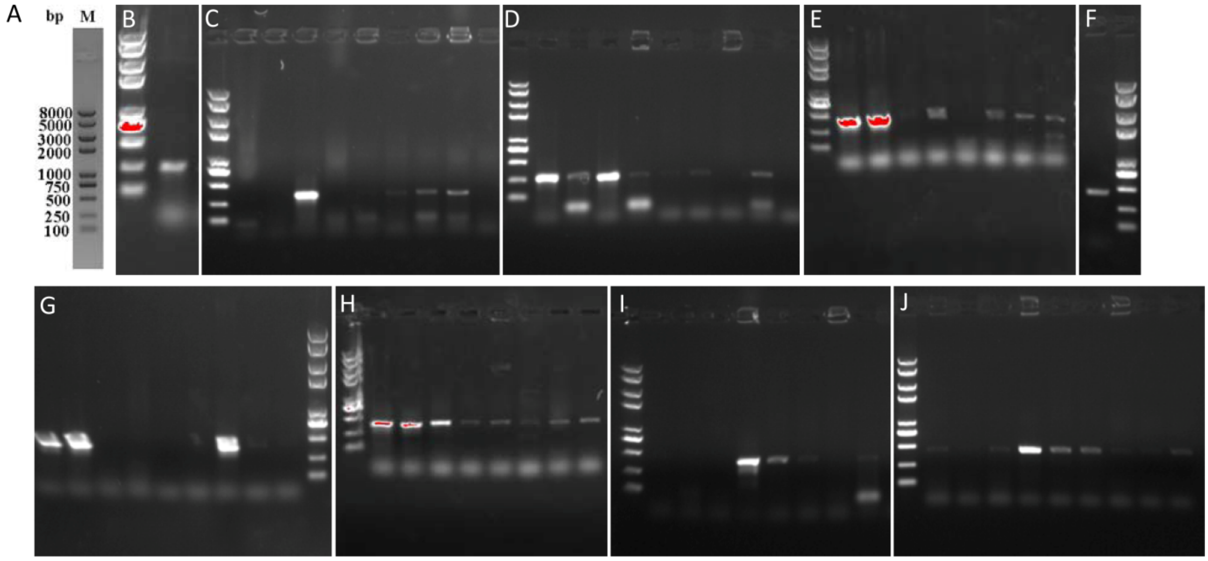

Supplementary Figure S10 The profiles of the enterotoxin gene types of staphylococcal isolates used in this study. (A) Standard DNA marker used in the assay. (B) PCR detection of enterotoxin c. Lanes (from left to right): Marker, WHS11090. (C-D) PCR detection of enterotoxin d. (C) lane 1: Marker, lane 4: WHS11089; lanes 7-9: WHS11091, WHS11080, WHS11081; (D) Lanes (from left to right): WHSWHS11082, WHS11083, WHS11086, WHS11071, WHS11072, WHS11075. (E) PCR detection of enterotoxin g. Lane1: Marker; lanes 2-3: WHS11089, WHS11090; lane 5: WHS11091; lanes 7-9: WHS11092, WHSWHS11086, WHS11093. (F) PCR detection of enterotoxin j. Lane 1: WHS11075; lane 2: marker. (G) PCR detection of enterotoxin g. Lanes 1-2: WHS11085, WHS11094; lane 7: WHS11076. (H-I) PCR detection of enterotoxin i. (H) Lanes (from left to right): Marker, WHS11089, WHS11090, WHS11091, WHS11092, WHS11084, WHS11093, WHS11085, WHS11086. (I) lane 1: Marker, lanes 5-7: WHS11094, WHS11072, WHS11075; lane 9: WHS11076. (J) PCR detection of enterotoxin j. Lanes 1-2: marker, WHS11080; Lanes 4-10: WHS11081, WHS11082,WHS1083,WHS11092, WHS11086, WHS11072, WHS11077.

Supplementary References (for Supplementary Table S1)

1 Towner, K. J., Talbot, D. C., Curran, R., Webster, C. A. & Humphreys, H. Development and evaluation of a PCR-based immunoassay for the rapid detection of methicillin-resistant *Staphylococcus aureus*. *J Med Microbiol* **47**, 607-613 (1998).

2 Mehrotra, M., Wang, G. & Johnson, W. M. Multiplex PCR for detection of genes for *Staphylococcus aureus* enterotoxins, exfoliative toxins, toxic shock syndrome toxin 1, and methicillin resistance. *J Clin Microbiol* **38**, 1032-1035 (2000).

3 Gocmen Julide, S., Sahiner, N., Kocak, M. & Karahan, Z. C. PCR investigation of Panton-Valentine leukocidin, enterotoxin, exfoliative toxin, and agr genes in *Staphylococcus aureus* strains isolated from psoriasis patients. *Turk J Med Sci* **45**, 1345-1352 (2015).

4 Boye, K., Bartels, M. D., Andersen, I. S., Moller, J. A. & Westh, H. A new multiplex PCR for easy screening of methicillin-resistant *Staphylococcus aureus* SCCmec types I-V. *Clin Microbiol Infect* **13**, 725-727 (2007).
